# Detection and Significance of Cytotoxic Cell Subsets in Biopsies of HCV-Infected Human Livers

**DOI:** 10.1007/s00005-013-0258-6

**Published:** 2013-11-14

**Authors:** Iwona Mozer-Lisewska, Anna Mania, Arleta Kowala-Piaskowska, Andrzej Kluk, Husam Samara, Anna Pauli, Jan Żeromski

**Affiliations:** 1 Chair and Department of Infectious Diseases, Karol Marcinkowski University of Medical Sciences, Poznan, Poland; 2 Department of Pediatric Infectious Diseases and Child Neurology, Karol Marcinkowski University of Medical Sciences, Poznan, Poland; 3 Department of Clinical Pathomorphology, Karol Marcinkowski University of Medical Sciences, Poznan, Poland; 4 Department of Clinical Immunology, Karol Marcinkowski University of Medical Sciences, Poznan, Poland

**Keywords:** HCV^+^ liver biopsy, NK cells, Cytotoxic T cells, NKG2D receptor, Immunohistochemistry, Patients data

## Abstract

Chronic viral hepatitis C still remains the clinical challenge. Attempts of the immune system to cope with this infection are unsatisfactory. There is a conviction that the main site of interaction between virus (Hepatitis C virus, HCV) and immune system is in situ, i.e., in liver. Natural killer (NK) cells appeared relevant in the acute hepatitis. Less is known about the immune response in the chronic HCV infection. The aim of this study was to evaluate the prevalence of various cytotoxic cell subsets in chronic HCV^+^ liver tissue and to seek links between them and laboratory data of patients. Sections from paraffin blocks of liver biopsy tissues of HCV^+^ untreated patients were subjected to the reaction with antibodies vs. cytotoxic cell subsets and immunohistochemistry. Positive cells were searched in cellular infiltrates in portal areas and in liver parenchyma. They were classified on the “Yes” or “No” basis. Majority of liver biopsies exhibited cellular infiltrates in portal spaces and as single cells in liver parenchyma. Infiltrates consisted of CD8^+^ T cells, CD56^+^ NK ones, including CD158i^+^ and CD158b^+^. The latter were rarely seen. There were also granzyme B^+^ cells. The most abundant were NKG2D^+^ cells, much more common than NK CD56^+^ ones. It implied that NKG2D was also expressed on T cells. Prevalence of NKG2D^+^ cells correlated with high activity of liver enzymes such as alanine aminotransferase, aspartate aminotransferase and a greater histological severity of liver injury. NKG2D^+^ cells form the bulk of cells infiltrating HCV-infected human liver. Correlation of NKG2D^+^ cells with some laboratory parameters of patients suggests their role in hepatitis C pathogenesis.

## Introduction

Chronic viral hepatitis C remains one of the most challenging human diseases. It affects almost 200 million people worldwide. Inflammatory processes lead to severe liver damage manifested by fatty change, cell infiltrates, hepatocyte apoptosis, culminating in liver cirrhosis and finally hepatocellular carcinoma. As specific vaccine is still unavailable, hepatitis C virus (HCV) infection spreads out by various parenteral routes worldwide, particularly in developing countries (Rantala and van de Laar 2008; Thomas and Seeff 2005).

The host immune system has only limited tools to control HCV entry into the body. Infected cells secrete interferon type I (α) that has a virostatic effect and partly prevents infection of surrounding cells. Natural killer (NK) cells are the only ones of innate immunity, able to kill cells already infected by the virus in question. They possess the ability to discern self from non-self, either by the recognition of viral peptides on the cell surface, or by down-regulation or loss of some MHC molecules (Lodoen and Lanier 2006). The latter happens mainly in cancer, but also in viral invasions and affects various HLA A–C alleles. This peculiar feature of NK cells is due to the expression on their surface of several sets of receptors such as immunoglobulin-like ones (KIR), lectin-like ones, natural cytotoxic ones and others. Receptor ligands are various HLA alleles, both polymorphic and non-polymorphic but also some viral antigens, for example, influenza hemagglutinin. Receptors are either inhibitory or activating, depending on the recognized ligand (Bryceson et al. 2011). It has been reported that the reduction of expression of some NK receptors in recently HCV-infected patients may be linked to virus clearance (Alter et al. 2011). Induction of apoptosis of HCV-infected hepatocytes by NK cells has been shown in acute phase of hepatitis C, manifested occasionally by the inhibition of viral spread (Golden-Mason et al. 2010; Zou et al. 2010). Relatively little, however, is known about the function and biological significance of NK cells in chronic phase of this disease, in spite of considerable body of research on this disease (Brenndorfer and Sallberg 2012; Zeromski et al. 2011). Several authors have investigated other lymphoid cell subsets of liver infiltrates, such as T cells, B cells, etc. (Fiore et al. 1997; Koziel and Walker 1997). T-regulatory cells were extensively studied by various groups and considered to be involved in the induction of tolerance to viral antigens in affected hosts (Amoroso et al. 2012; Miyaaki et al. 2009). On the contrary, NK cells were found to be suppressed by HCV proteins (Cheent and Khakoo 2011; Varchetta et al. 2012; Wen et al. 2008). Their frequencies in peripheral blood are reduced in chronic HCV infections (Cheent and Khakoo 2011). It has been postulated that direct cell-to-cell contact of NK cells with infected hepatocytes inhibits cytotoxicity of these cells (Yoon et al. 2011). Recently however, it has been shown in animal studies that mice (depleted of NK cells) infected with lymphocytic choriomeningitis virus (LCMV) are able to mount efficient adaptive CD8^+^ T-cell response against LCMV (Lang et al. 2012). Thus, a possible regulatory role for NK cells in chronic viral infections has emerged and deserves investigation. Relatively little is known about another cell subset, namely natural killer T (NKT) cells (CD56^+^, CD3^+^), constituting 5–10 % of human liver lymphocytes. They recognize lipid antigens presented by the non-classical MHC class I-like molecules CD1. Most of them possess an invariant T-cell receptor α. When activated, NKT cells can produce large quantities of several cytokines such as IFN-γ, IL-17, TNF-α and others, as well as cytotoxic mediators including Fas ligand and TRAIL. Their activating ligands are unknown, making their functional utility elusive (Gao et al. 2009).

The aim of this study was to search for NK cells, some of their subsets as well as T cells in situ by immunohistochemistry (IHC) in tissues of diagnostic liver biopsies collected from HCV-infected patients prior to anti-viral therapy and compare these data to various clinical/laboratory parameters of individual patients. It was expected that such information might have some predictive value. It will be shown that some relationships do exist.

## Materials and Methods

### Patients

Patients were routinely admitted to the Hepatology Policlinic of Poznan J. Strus Municipal Hospital with suspected HCV infection. When registered, they signed written informed consent for their thorough diagnostic procedures and anti-viral therapy. They were submitted to comprehensive clinical and laboratory examinations, including the determination of anti-HCV antibodies, quantitative RNA-HCV viral load (Amplicor Monitor Test, Version 2.0—Roche, detection limit 50 IU/mL), alanine aminotransferase (ALT), aspartate aminotransferase (AST), γ glutamyltransferase liver enzymes activity, bilirubin content, α-fetoprotein and percutaneous liver biopsy. All procedures were in agreement with ethical guidelines of the 1975 Declaration of Helsinki and were approved by the Medical School Bioethics Committee.

#### Patient’s Tissues

Paraffin blocks with embedded liver biopsy fragments from 56 HCV^+^ patients were used. Liver biopsy was routinely performed before anti-viral therapy. Hematoxylin and eosin (H + E) stained 4 μm sections, cut from paraffin liver tissue blocks, were evaluated for grading and staging utilizing METAVIR (1994) scale.

### Immunohistochemistry

Formalin fixed, paraffin wax embedded liver specimens were cut in 4 μm sections. The latter were mounted on adhesive microscopic slides (Super FrostPlus, Menzel Glaser), dewaxed with xylene, and dehydrated in decreasing content of alcohol. Antigen retrieval was obtained by heating in a water bath (buffer-Epitope Retrieval Solution, pH 9, Novocastra Leica Microsystems) for 50 min at 98 °C. Endogenous peroxidase was blocked by 3 % hydrogen peroxide for 20 min and thereafter sections were then incubated with primary antibody in a humidified chamber at 4 °C overnight. The primary antibodies and their concentrations used are depicted in Table 1. Primary antibodies were diluted using IHC Diluent (Leica Microsystems). For each section, a negative control was performed. Instead of primary antibody, IHC Diluent was applied. As positive controls, the following tissue sections were used: for granzyme B—Hodgkin’s lymphoma, for CD56—large bowel, for NKG2D—CD158i and CD158b hyperplastic lymph node. The immunoreaction was localized using the NovoLink Polymer Detection System (Leica Microsystems). It is a two-step streptavidin–biotin-peroxidase method. Each slide was incubated for 30 min at room temperature. Between reaction steps, slides were washed thrice in Tris-buffered saline (pH 7.6) for 5 min. Visualization of reaction was achieved by 3,3′-diaminobenzidine tetrachloride (DAB) (Leica Microsystems). Sections were counterstained with Mayer’s hematoxylin, dehydrated, cleared and mounted in Canada balsam.

**Table 1:** Primary antibodies used

Specificity	Animal	Type	Clone	Source	Catalog number
CD56/NCAM	Mouse	Monoclonal	CD564	Leica Microsystems	NCL-CD56-504
CD3	Rabbit	Polyclonal	NS	Dako	IR503
CD8	Mouse	Monoclonal	1A5	Leica	NCL-CD8-295
NKG2D/CD314	Rabbit	Polyclonal	KLRK1	Bioss Inc.	bs-0938R
NKG2D/CD314	Mouse	Monoclonal	1D11	Novus Biologicals	NB100-65956
Granzyme B	Mouse	Monoclonal	GZB01	Abcam	Ab3654
KIR2DS4/CD158i	Rabbit	Polyclonal	NS	Bioss Inc.	Bs-2644R
KIR2DL2/CD158b	Rabbit	Polyclonal	NS	Bioss Inc.	Bs-2642R

*NS* not shown

#### Evaluation of IHC Reactions

Slides were evaluated by competent, experienced immunopathologist (JZ) using a research light microscope (Olympus BX40), with 40× objective (magnification 400×), and five high-power fields were searched for:

- the presence of positive cells in portal areas and/or in liver parenchyma, and
- positive cells in cell clusters and/or single dispersed ones.

### Statistical Analysis

It was performed using Statistica StatSoft package. Analyzed data was not normally distributed. The parametric data comparisons were made by Mann–Whitney *U* test. Nonparametric data were compared using Fisher’s test, the Chi-square test and Spearman rank correlation test. Continuous variables were tested for normality using Shapiro–Wilk test and compared using Mann–Whitney *U* test. Categorical variables were tested using Fisher’s exact test or Chi-square test, where appropriate. Correlations were performed using Spearman rank correlation test. Univariate analysis was performed on chosen parameters. Significant parameters were included in multivariate analysis. Values were considered statistically significant if *p* < 0.05 or confidence interval did not include 1.

## Results

**Fig. 1:**
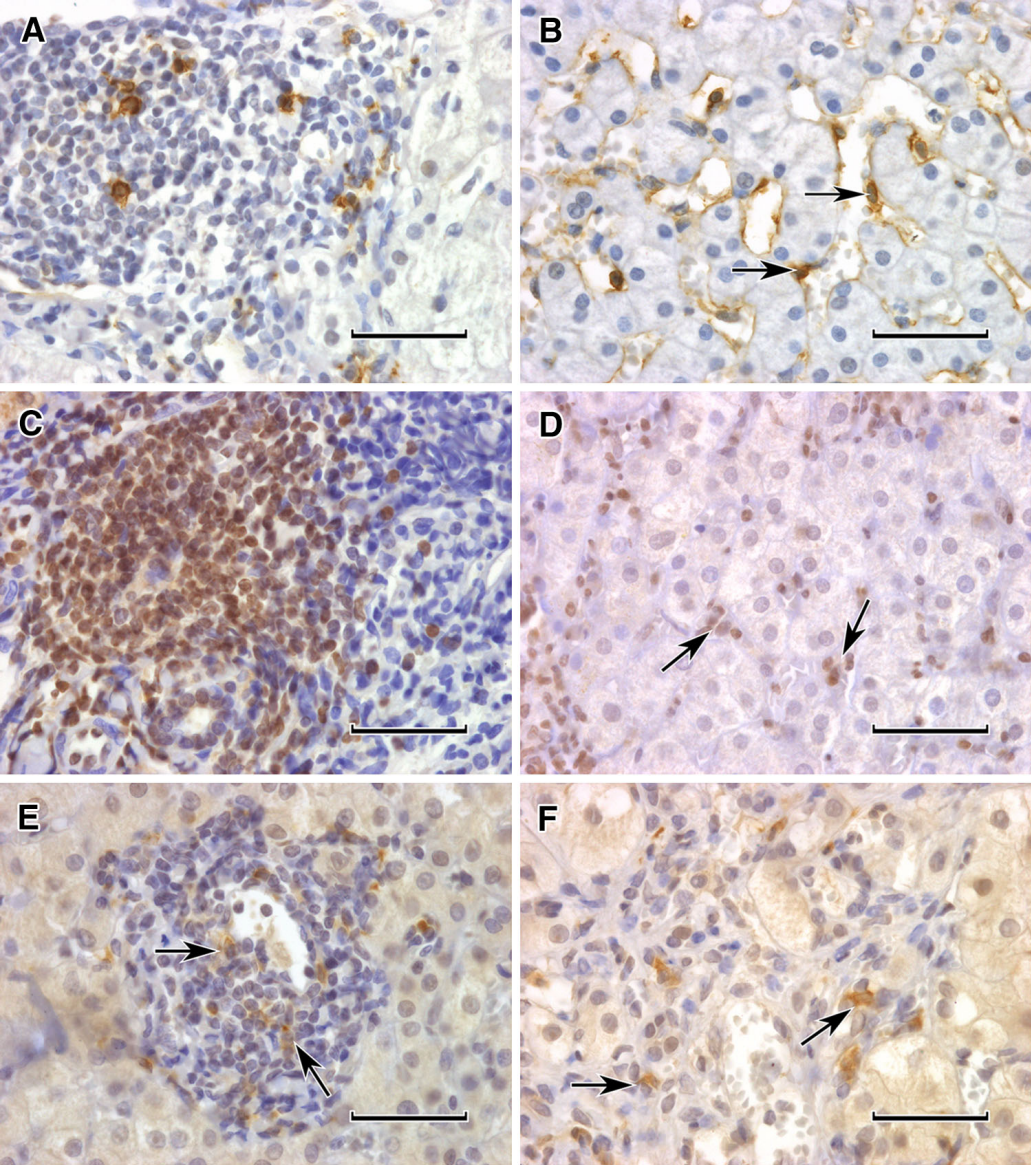
Immunohistochemical pictures of NK cells in HCV-infected liver tissues (1–6). **a** Single positive cells in cellular infiltrate of portal area, anti-CD56 monoclonal antibody (MoAb), ABC immunohistochemistry (IHC) reaction. **b** Single positive cells in liver parenchyma (*arrows*), anti-CD56 MoAb, the reaction as previously. **c** Abundant positive cells in cellular infiltrate of portal area, anti-NKG2D MoAb, ABC IHC. **d** Single positive cells in liver parenchyma (*arrows*), the reaction as previously. **e** Single positive dispersed cells in liver cellular infiltrate (*arrows*), anti-CD158i NK antibody (Ab), ABC IHC. **f** Single positive cells in liver cellular infiltrate (*arrows*), anti-CD158b NK Ab, ABC IHC. *Bar in all* 50 μm

**Fig. 2:**
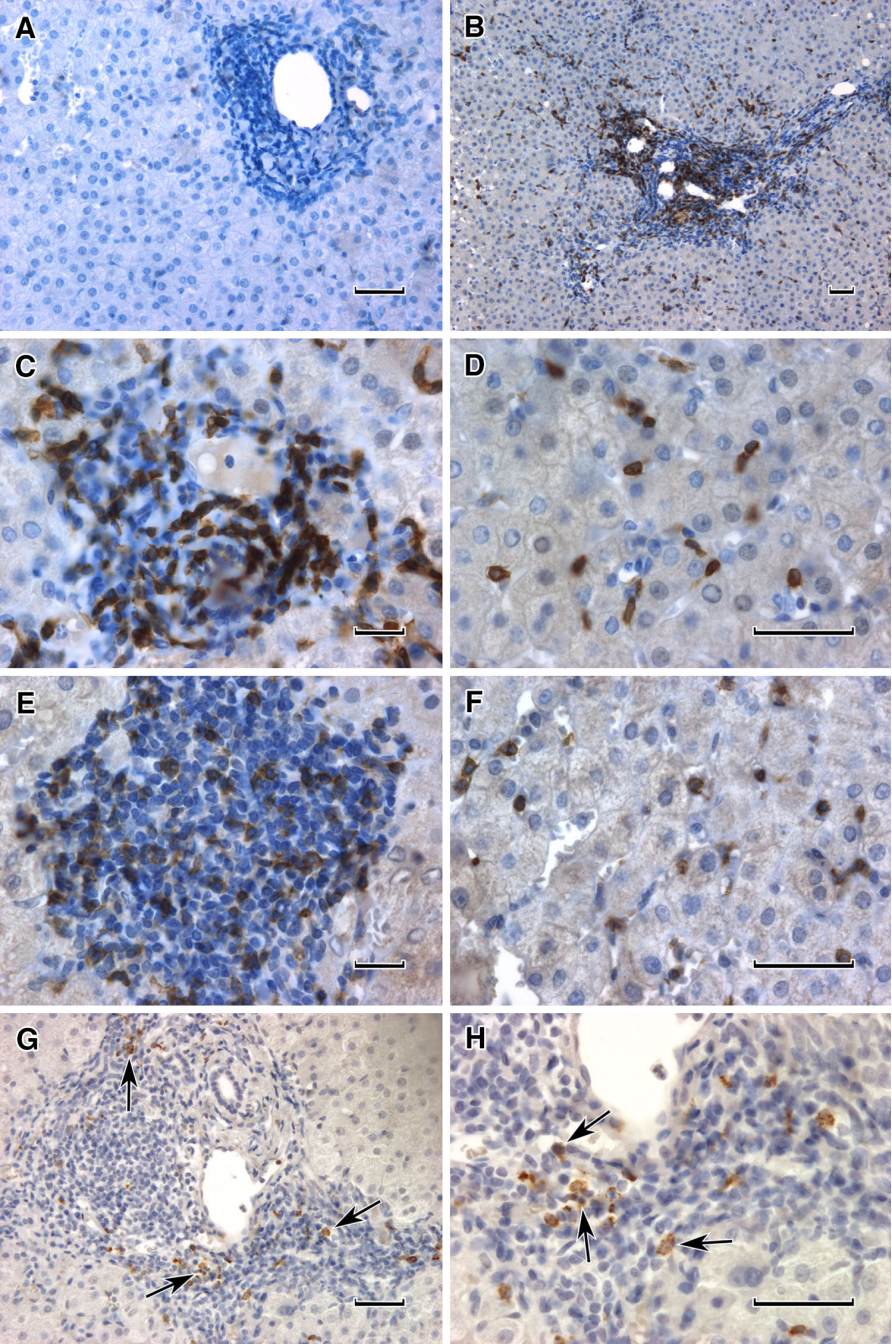
Immunohistochemical pictures of T-cell subsets and granzyme B in HCV-infected liver tissues (1–8). **a** Visible abundant cellular infiltrate in portal area, control reaction, PBS instead of primary antibody followed by ABC IHC, H + E counterstain. **b** Positive cells visible both in cellular infiltrate of portal area and in liver parenchyma, anti-T CD3 Ab, ABC IHC. **c** As previously but only portal area. **d** As previously but liver parenchyma, visible single positive cells. **e** Abundant dense cellular infiltrate, numerous positive cells visible, anti-T CD8 MoAb, ABC IHC. **f** As previously but liver parenchyma, visible single dispersed positive cells. **g** Intensive cellular infiltrate in portal area, single dispersed positive cells visible (*arrows*), anti-granzyme B MoAb. **h** As previously but high power (*arrows*). *Bar in all* 50 μm

Frequency of detection of positive cells either in singles or in clusters is shown in Table 2. It can be seen that NKG2D^+^ cells were most frequently observed, predominantly in portal areas. It was also true for the remaining NK cells such as CD56^+^, CD158i^+^ and CD158b^+^ ones. Granzyme B^+^ cells were also mainly seen within portal spaces. NK^+^ cells were only rarely seen as single ones dispersed in liver parenchyma. On the contrary, T cells (both CD3^+^ and CD8^+^) apart from portal spaces were frequently seen between hepatocytes (Figs. 1, 2).

**Table 2:** Prevalence of positive cells in IHC reactions

Antibody vs.	Single positive cells	Positive cell clusters	Lack of positive cells
CD56	32	16	8
Granzyme B	24	11	21
NKG2D	7	36	13
CD158i	20	22	14
CD158b	9	4	43

Total biopsies tested: 56

There was no correlation among CD56-positive cells and the results of histopathology, viral load and biochemical assays. The observed differences were not statistically significant. The expression of NK cell receptors, CD158i and 158b also did not correlate significantly with deviations in biochemistry (Tables 3, 4).

**Table 3:** Biochemical parameters, viral load, histology of biopsy tissue in a studied group depending on NKG2D expression on cellular infiltrates

Parameter	Patient’s subgroup with NKG2D expression (*n* = 43)	Patient’s subgroup without NKG2D expression (*n* = 13)	*p*
Viremia log 10	4.40 ± 0.95	4.58 ± 0.99	0.565
ALT>Y/N (normal limit up to 40 IU/L)	30/13	5/8	0.044*
ALT before treatment	67.03 ± 41.22	39.39 ± 24.47	0.012*
AST>Y/N (normal limit up to 42 IU/L)	18/25	3/10	0.856
AST before treatment	42.00 ± 21.77	28.96 ± 11.59	0.014*
AFP	7.29 ± 13.25	6.28 ± 11.27	0.199
GGTP (normal limit up to 60 U/L)	43.74 ± 32.95	37.62 ± 21.03	0.819
Grading	1.67 ± 0.71	1.15 ± 0.55	0.019*
Staging	1.744 ± 1.00	1.1 ± 0.48	0.136
Prevalence of steatosis Y/N	18/25	2/11	0.075
Percent of steatosis	10.37 ± 19.75	2.54 ± 5.67	0.71
Bilirubin level	0.86 ± 0.56	0.96 ± 0.52	0.317

*AFP* α-fetoprotein, *GGTP* γ glutamyltransferase, *Y/N* yes/no

* *p* < 0.05

**Table 4:** Relationship between in situ cell NKG2D expression and patient’s laboratory parameters

Factor	Univariate analysis		Multivariate analysis	
	OR (95 % CI)	*p*	OR (95 % CI)	*p*
Log 10 HCV-RNA	1.22 (0.60–2.50)	0.558	**–**	
ALT>UNL Y/N	0.27 (0.07–1.01)	0.047*	**–**	
ALT IU/L before treatment	0.97 (0.94–0.99)	0.027*	0.98 (0.93**–**1.02)	0.371
AST>UNL Y/N	0.42 (0.09–1.79)	0.228	**–**	
AST IU/L before treatment	0.94 (0.88–0.99)	0.038*	0.98 (0.89**–**1.09)	0.838
Bilirubin mg/dL before treatment	1.38 (0.45–4.15)	0.570	**–**	
AFP before treatment	0.99 (0.93–1.05)	0.801	**–**	
GGTP IU/L	0.99 (0.97–1.02)	0.543	0.46 (0.12**–**1.69)	0.232
Grading	0.29 (0.09–0.89)	0.028*	**–**	
Staging	0.55 (0.24–1.24)	0.141	**–**	
Steatosis Y/N	0.25 (0.05–1.33)	0.097	**–**	

*AFP* α-fetoprotein, *GGTP* γ glutamyltransferase, *UNL* upper normal limit, *OR* odd ratio, *CI* confidence interval, *Y/N* yes/no

* *p* < 0.05

**Fig. 3:**
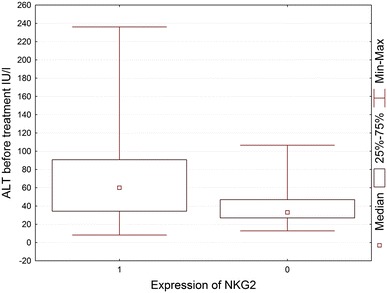
Median ALT activity before anti-viral treatment in children with and without expression of NKG2 in the liver biopsy specimens

**Fig. 4:**
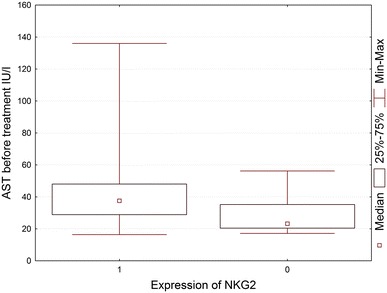
Median AST activity before anti-viral treatment in children with and without expression of NKG2 in the liver biopsy specimens

**Fig. 5:**
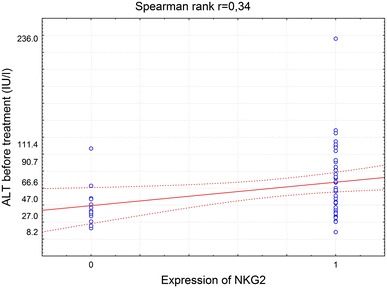
Spearman rank correlation between ALT activity and NKG2 expression in liver biopsy specimens

**Fig. 6:**
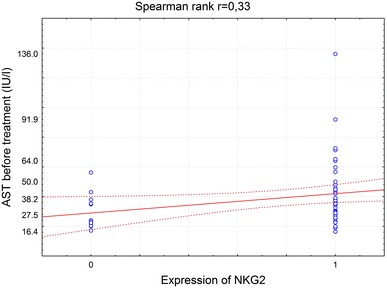
Spearman rank correlation between AST activity and NKG2 expression in liver biopsy specimens

**Fig. 7:**
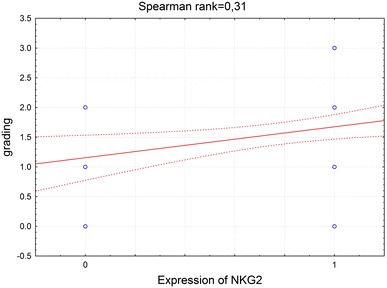
Spearman rank correlation between scores of histological grading and cell NKG2 receptor expression in liver biopsy specimens

In order to investigate the correlation between the most frequently seen NKG2D^+^ cells in HCV^+^ liver and clinical parameters of patients, NKG2D cell expression was compared to the patients laboratory data (Tables 3, 4). It was found that NKG2D expression on cells infiltrating HCV^+^ liver showed a correlation with the rise of activity of liver enzymes, ALT and AST (Figs. 3, 4, 5, 6). There was also a correlation between the histological grading and the intensity of liver steatosis and the prevalence of NKG2D^+^ cells (Fig. 7).

## Discussion

The results of the present study indicate that cellular infiltrates in HCV^+^ human liver are quite complex and consist of several cell types in various proportions. We were initially mainly interested in screening NK cells and their markers related to their cytotoxic properties in virally infected liver. It turned out that other cell subsets have to be taken into account while looking for cells able to eliminate infected hepatocytes. Such cells were most abundant in portal areas, forming dense cellular infiltrates. They, however, could be also seen as single dispersed ones, in liver parenchyma between hepatocytes. In general, NK cells represented by CD56^+^ or CD158i^+^ were predominantly seen in cellular infiltrates, while only rarely spotted between liver cells. On the contrary, T cells, CD3^+^ or CD8^+^ ones, apart from strong representation in cell infiltrates, were quite common in liver parenchyma. This finding is unexplained, but suggests that effector cells of adaptive immunity such as CD8^+^ T cells are presumably more efficient in eliminating the infected hepatocytes than NK cells in the chronic phase of HCV infection (Lauer et al. 2002). It has been found that intrahepatic CD8^+^ T cells show higher NKG2D expression than circulating CD8^+^ T ones (Kennedy et al. 2008). All patients in the present study were yet to undergo anti-viral therapy, meaning that the observed findings are exclusively the host’s immune system attempts to cope with HCV infection.

An interesting observation was the abundance of NKG2D^+^ cells in cellular infiltrates predominantly in portal areas. Their density was much stronger than that of CD56^+^ cells, implying that other cells presumably T cells express NKG2D, apart from NK ones. The expression of NKG2A inhibitory receptor by activated T cells in chronic hepatitis C was shown by Nattermann et al. (2006). NKG2D (CD314) is an activating receptor but belongs to the same C-type lectin family of NK receptors as NKG2A. It is expressed on NK cell subset and memory T cells (Ahlenstiel and Rehermann 2007). Its ligands are stress-inducible MICA and MICB, MHC class I-like proteins, usually expressed in low quantities on epithelial cells (in particular, of gastrointestinal tract) but upregulated on infected ones, sufficient to activate NK cell receptors such as NKG2D (Delves et al. 2011). This may be the case in chronic hepatitis C. At any rate, NKG2D^+^ cells were the most frequently seen NK cell receptors tested in the present study. The specificity of IHC reaction was confirmed by the use of two distinct (monoclonal and polyclonal) antibodies of anti-NKG2D receptor. Thus, it seemed of interest to compare their expression with clinical parameters of patients. When mean values of individual factors of HCV^+^ patients were related to mean NKG2D-positive and NKG2D-negative liver biopsies, some relationships were found to be significant. It was evident for both ALT and AST liver enzymes, and histological grading that high values of these parameters correlated with the relative abundance of NKG2D^+^ cells in liver tissues. This was also confirmed in univariate but not in multivariate analysis. It suggests that cells expressing NKG2D activating receptor, both NK and T cells, participate in hepatitis C pathogenesis, apparently manifesting cytotoxic potential.

## Abbreviations

ALT: Alanine aminotransferase
AST: Aspartate aminotransferase
CD: Cluster determinant
HCV: Hepatitis C virus
IHC: Immunohistochemistry
NK: Natural killer
NKT: Natural killer T cells
